# Regional genetic differences among Japanese populations and performance of genotype imputation using whole-genome reference panel of the Tohoku Medical Megabank Project

**DOI:** 10.1186/s12864-018-4942-0

**Published:** 2018-07-24

**Authors:** Jun Yasuda, Fumiki Katsuoka, Inaho Danjoh, Yosuke Kawai, Kaname Kojima, Masao Nagasaki, Sakae Saito, Yumi Yamaguchi-Kabata, Shu Tadaka, Ikuko N. Motoike, Kazuki Kumada, Mika Sakurai-Yageta, Osamu Tanabe, Nobuo Fuse, Gen Tamiya, Koichiro Higasa, Fumihiko Matsuda, Nobufumi Yasuda, Motoki Iwasaki, Makoto Sasaki, Atsushi Shimizu, Kengo Kinoshita, Masayuki Yamamoto

**Affiliations:** 1 Sendai, Tohoku Medical Megabank Organization, 2-1, Seiryo-machi, Aoba-ku, Tohoku Medical Megabank, Tohoku University, Sendai, 980-8573 Miyagi Japan; 2 Center for Genomic Medicine, Kyoto University Graduate School of Medicine, Kyoto, 606-8501 Sakyo-ku Japan; 3 Department of Public Health, Kochi University Medical School, Nankoku-shi, 783-8505 Kochi Japan; 4 Division of Epidemiology, Center for Public Health Sciences, National Cancer Center, Tokyo, 104-0045 Chuo-ku Japan; 5 Division of Ultrahigh Field MRI, Institute for Biomedical Sciences, Iwate Medical University, 2-1-1 Nishitokuta, Yahaba, Shiwa, Iwate, 028-3694 Japan; 6 Iwate Tohoku Medical Megabank Organization, Disaster Reconstruction Center, Iwate Medical University, 2-1-1 Nishitokuta, Yahaba, Shiwa, Iwate, 028-3694 Japan; 7 Graduate School of Information Sciences, Tohoku University, Aoba-ku, Sendai, 980-8579 Miyagi Japan; 8 Department of Medical Biochemistry, Graduate School of Medicine, Tohoku University, Aoba-ku, Sendai, 980-8575 Miyagi Japan; 9 Present address: Department of Human Genetics, Graduate School of Medicine, The University of Tokyo, Bunkyo-ku, 113-0033 Tokyo Japan

**Keywords:** Genome reference panel, Genotype imputation, Population genetics, Japan

## Abstract

**Background:**

Genotype imputation from single-nucleotide polymorphism (SNP) genotype data using a haplotype reference panel consisting of thousands of unrelated individuals from populations of interest can help to identify strongly associated variants in genome-wide association studies. The Tohoku Medical Megabank (TMM) project was established to support the development of precision medicine, together with the whole-genome sequencing of 1070 human genomes from individuals in the Miyagi region (Northeast Japan) and the construction of the 1070 Japanese genome reference panel (1KJPN). Here, we investigated the performance of 1KJPN for genotype imputation of Japanese samples not included in the TMM project and compared it with other population reference panels.

**Results:**

We found that the 1KJPN population was more similar to other Japanese populations, Nagahama (south-central Japan) and Aki (Shikoku Island), than to East Asian populations in the 1000 Genomes Project other than JPT, suggesting that the large-scale collection (more than 1000) of Japanese genomes from the Miyagi region covered many of the genetic variations of Japanese in mainland Japan. Moreover, 1KJPN outperformed the phase 3 reference panel of the 1000 Genomes Project (1KGPp3) for Japanese samples, and IKJPN showed similar imputation rates for the TMM and other Japanese samples for SNPs with minor allele frequencies (MAFs) higher than 1%.

**Conclusions:**

1KJPN covered most of the variants found in the samples from areas of the Japanese mainland outside the Miyagi region, implying 1KJPN is representative of the Japanese population’s genomes. 1KJPN and successive reference panels are useful genome reference panels for the mainland Japanese population. Importantly, the addition of whole genome sequences not included in the 1KJPN panel improved imputation efficiencies for SNPs with MAFs under 1% for samples from most regions of the Japanese archipelago.

**Electronic supplementary material:**

The online version of this article (10.1186/s12864-018-4942-0) contains supplementary material, which is available to authorized users.

## Background

Genotype imputation is an important step in current genome-wide association studies. Imputation accuracy, as well as genomic coverage of highly accurate imputed genotypes, confers elevated statistical power in association tests. [1] The choice of a haplotype reference panel to maximize imputation performance has often been debated. [2–4] Haplotype reference panels are used to identify haplotypes of individual genomes genotyped by single-nucleotide polymorphism (SNP) arrays, and then to estimate the genotypes missing in the SNP array data. Thus, to enable high-density genotype imputation for SNPs with minor allele frequencies (MAFs) > 1% in a population, reference panels are constructed preferably based on the whole-genome sequencing (WGS) of large samples. The influence of panel selection on imputation accuracy in terms of panel size and ancestry matching between the panel and study samples has been assessed by cross-validation. [5] The results showed that better imputation performances were achieved when more samples from various populations were included in the reference panel. Thus, along with improved algorithms for genotype imputation using large panels, great efforts are being made to construct large reference panels for highly accurate genotype imputation, such as that by the Haplotype Reference Consortium. [4] In addition, several cohort studies have been conducted using WGS to construct better, more detailed haplotype reference panels. [2, 6] These studies suggest that increasing the sample sizes of population-specific haplotype reference panels is more effective for improving genotype imputation accuracy than aggregating the haplotype collection from worldwide resources, because the focus is then on specific populations. Although recent studies in human population genetics have revealed clear regional variation in haplotype diversity, even within a single population, [7] the influence of such variation on imputation performance has not yet been assessed.

The Tohoku Medical Megabank (TMM) project was launched in 2011 to investigate effects in the aftermath of the Great East Japan Earthquake in the Miyagi and Iwate prefectures (Northeast Japan). The TMM project developed prospective cohorts in these two prefectures [8] with the aim of aiding in the establishment of precision medicine in this region. To contribute to this, the WGS of 1000 human genomes from individuals in the Miyagi region was undertaken (beginning in 2015) and a 1070 Japanese genome reference panel containing haplotype information was constructed using this data (the 1KJPN panel). [9] In a previous study, we reported the imputation performance using the 1KJPN panel was better than the performance using the 1000 Genomes Project phase 1 panel [10]. We also designed a custom SNP array for a Japanese population (the Japonica array). [11]

The 1KJPN panel consists of haplotypes derived from a cohort of participants in the Miyagi Prefecture, which has approximately 5% of Japan’s total population. However, it is not known how much region-specific sampling for a reference panel affects the performance of genotype imputation for samples collected nationwide.

**Fig. 1:**
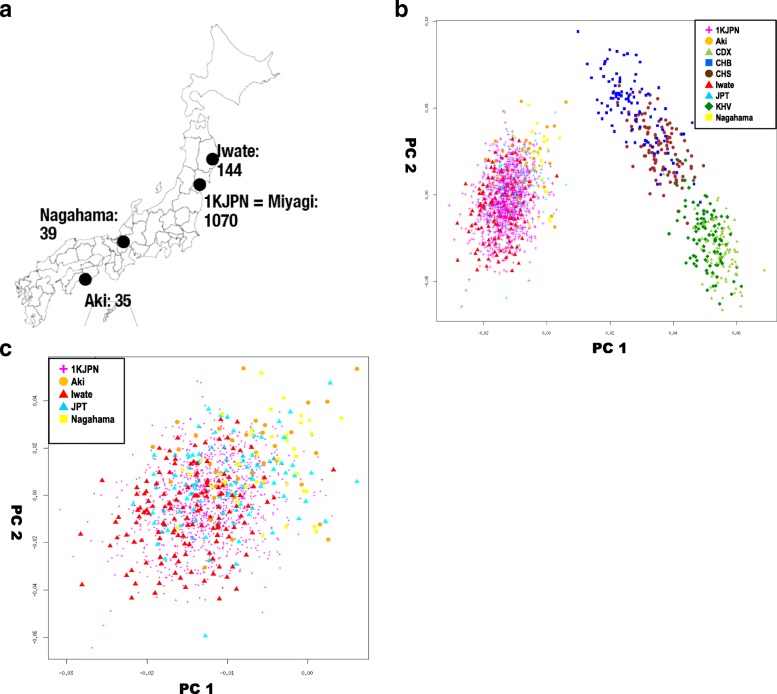
Human genomic similarities among four different regions of mainland Japan. **a**. Schematic diagram indicating the geographic origins of the Japanese samples used in this study. **b**. Scatter plot of first and second eigenvalues in principal component analysis (PCA) of genetic diversity in chromosome 1 of Japanese and other East Asian populations analyzed in the 1000 Genomes Project. Horizontal and vertical axes indicate the first and second components, respectively. The dots represent individuals and the legends are shown on the top right. C. PCA of the five Japanese populations analyzed in this study (close up view of Fig. 1b). The individuals used in the 1KJPN panel are indicated by small dots to improve the visualization of other populations

In this study, we evaluated the imputation performance of the 1KJPN panel for participants from three areas other than Miyagi, namely, Iwate, Nagahama (Kinki region, south-central Japan), and Aki (Shikoku Island) (Fig. 1a). We also extended the 1KJPN panel by adding haplotype information from the samples from these three other areas to assess imputation performance given the haplotype variations across the different areas.

## Results

### Customized reference panel construction

We performed WGS for all the available samples from Iwate, Nagahama, and Aki to construct an extended haplotype reference panel (1KJPN+ panel; Fig. 1a). After removing one Aki sample because of the cryptic relatedness of another member of the cohort, the 1KJPN+ panel consisted of 2560 haplotypes from 1280 samples (1070, 136, 39, and 35 samples from Miyagi, Iwate, Nagahama, and Aki, respectively). We also compared our panels with the phase 3 panel of the 1000 Genomes Project [12] (1KGPp3 panel).

### Genetic diversity of Japanese and other east Asian populations

We compared the diversity of Japanese populations (namely, the Miyagi cohort used to construct 1KJPN and other Japanese populations) with the diversity of populations from elsewhere in East Asia to determine how 1KJPN might reflect these populations. Principal component analysis (PCA) plots with 35,596 SNP genotypes (not indels) on chromosome 1 (see methods) are shown in Fig. 1b. The proportion of variance explained by the first and second principal components was 15.4 and 3.53%, respectively. Japanese individuals from Aki, Iwate, and Nagahama who were newly added to the dataset were clustered with the Miyagi population (= 1KJPN) but separated from other East Asian populations analyzed in the 1000 Genomes Project. Indeed, the Miyagi samples overlapped with most of the other Japanese populations, as shown in Fig. 1c, which is a magnified view of the Japanese populations in Fig. 1b. This indicates that the 1KJPN population is sufficiently similar to populations elsewhere in Japan and that 1KJPN can be used as genomic data representative of the whole Japanese population in mainland Japan.

### Genetic diversity in Miyagi and other parts of Japan

The genetic differentiation of samples in four parts of Japan was investigated in terms of fixation index (F_ST_) and haplotype sharing. Although the PCA did not provide sufficient resolution to separate the samples into the four discrete areas (Fig. 1c), the F_ST_ values were in good agreement with the geographic locations (Table 1). For instance, the F_ST_ value between Miyagi and Iwate, which are adjacent prefectures, was the smallest (0.000345) among all pairs of areas examined, which corresponds to the findings in a recent study. [13] No pair of F_ST_ values among the four regions exceeded the F_ST_ between the CHB (Han Chinese in Beijing) population in the 1000 Genome Project and our Japanese samples (F_ST_ = 0.00777; Additional file 1: Table S1), indicating that 1KJPN may be an appropriate reference panel for the population of mainland Japan.

**Table 1:** Fixation index (F_ST_) estimation* of genetic differentiation of samples in four parts of Japan

	Iwate	Nagahama	Aki
Miyagi	0.000345	0.000380	0.00154
Iwate	–	0.000876	0.00192
Nagahama		–	0.000920

*The F_ST_ values are based on the SNP_S_ in chromosome 1

**Fig. 2:**
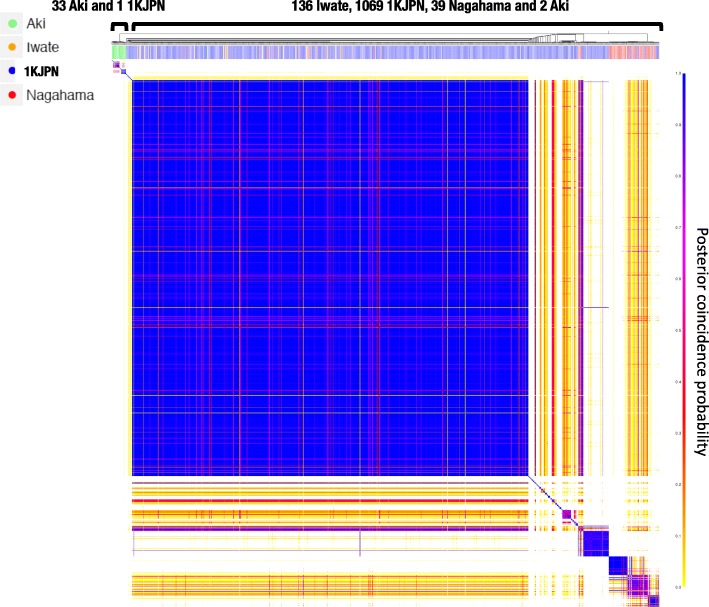
Pairwise coincidence matrix of individuals from the four Japanese populations created by fineSTRUCTURE. The color scale represents the posterior confidence probability. The origins of each sample are indicated with colors in the top left of the figure (different color codes compared with Fig. 1b and C). Four clusters are presented and the numbers of samples from each region are indicated in brackets at the top of the matrix

When we analyzed all the 1070 Miyagi samples using fineSTRUCTURE [14], the samples from Miyagi and Nagahama were assigned to the same cluster, whereas most of the samples from Aki formed a distinct cluster (Fig. 2). In this analysis the majority of Aki samples formed a distinct cluster from the other populations, indicating the Aki population from Shikoku Island may be somewhat distinct from the Honshu populations (Iwate, Miyagi, and Nagahama; Fig. 1a). These results show that, in Japan, a large collection of genomic DNA samples from a region the size of a prefectural (around 2% of the Japanese population) may have genetic diversity similar to that of the Japanese population as a whole.

Moreover, we chose 288 of the 1070 samples from Miyagi residents whose maternal grandmother was also born in Miyagi Prefecture to analyze haplotype sharing among the four Japanese regions using fineSTRUCTURE [14] (Additional file 2: Figure S1). We identified four clusters and found that the cluster positions corresponded to the geographic relationship among the regions. For example, cluster A consisted of samples from Iwate and Miyagi, which are adjacent prefectures, whereas cluster B was dominated by Miyagi samples with small numbers of Iwate and Nagahama samples. However, further investigation is needed to clarify whether the cluster separation among the regions, as shown in Additional file 2: Figure S1, comes from the simple reduction of analyzed individuals, the increase of the Miyagi-specific population, or both.

### 1KJPN genotype imputation efficiencies and effects of genetic differences

**Fig. 3:**
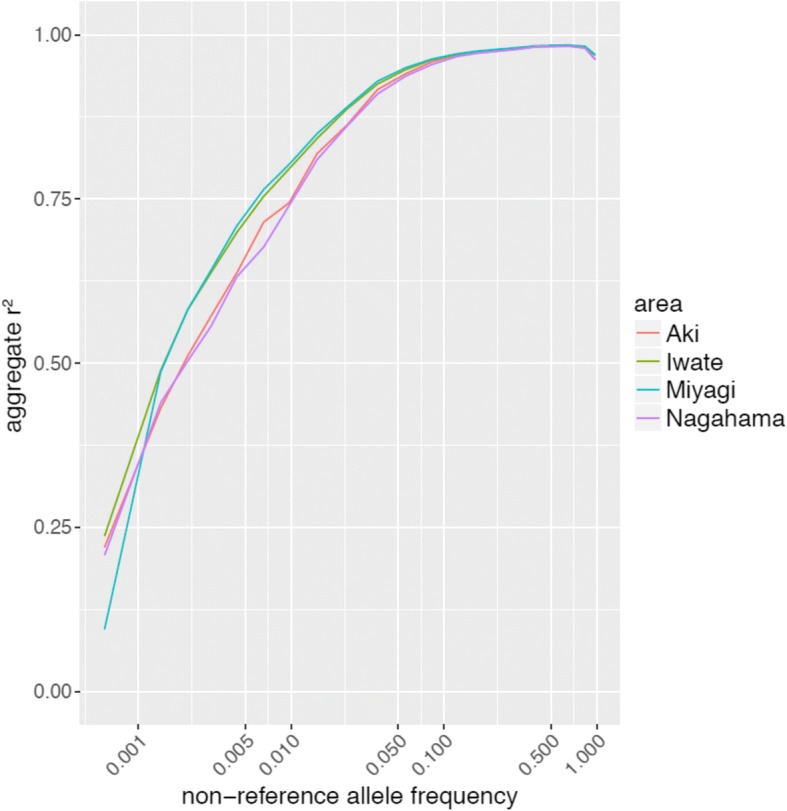
Imputation accuracies using 1KJPN data for Japanese populations not included in the 1KJPN panel. Plot of the imputation accuracy (vertical axis, aggregate r^2^ value) against the non-reference allele frequency of reference panel (horizontal axis) when the 1KJPN panel was used as the haplotype reference. Each population is indicated by a different color. Each point on the curves is the average of the corresponding allele frequency bin

To evaluate the difference in imputation performance of the samples from different regions, the aggregate of Pearson’s correlation coefficient (r^2^) values of imputed variants was compared among samples from different areas (Fig. 3). Imputation accuracies among samples from the four areas were comparable (r^2^ = 0.9891, 0.9881, 0.9875, and 0.9878 for Miyagi, Iwate, Nagahama, and Aki, respectively) for common SNPs (non-reference allele frequency > 5%), whereas the accuracies differed among samples from these areas for lower frequency variants. For example, for rare variants in 1KJPN (non-reference allele frequency ≤ 1%), the r^2^ values were 0.7123, 0.7067, 0.6312, and 0.6486 for Miyagi, Iwate, Nagahama, and Aki, respectively.

Genotype imputation is difficult for rare variants, so this decrease in aggregate r^2^ values was expected. However, SNPs with about 1% MAFs were efficiently imputed with 1KJPN for the Nagahama and Aki samples (Fig. 3; r^2^ values around 0.75). These results indicate that 1KJPN is adequate for use as a reference panel for populations from mainland Japan.

**Fig. 4:**
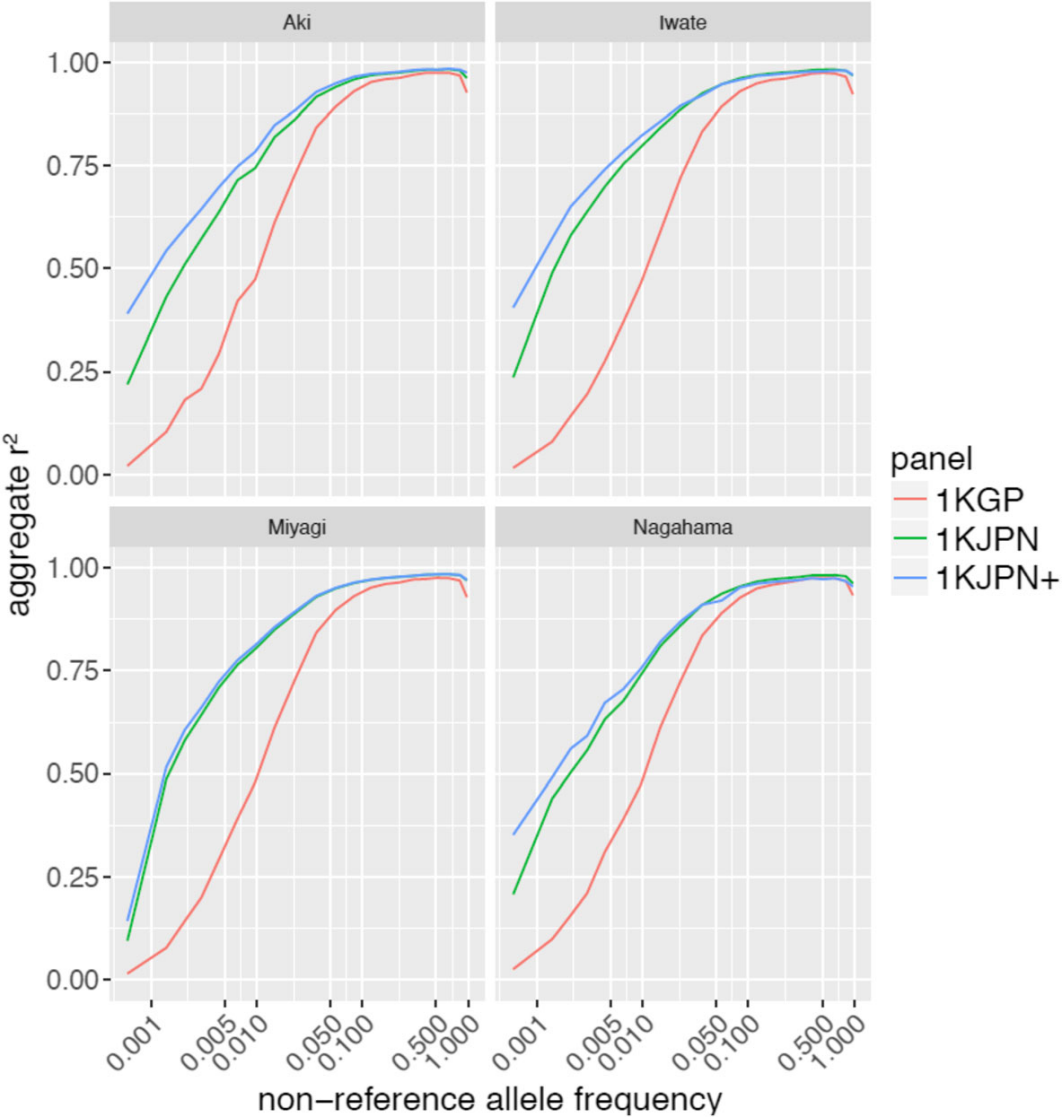
Differences in imputation accuracies using reference panels for Japanese populations. Vertical axis indicates the r^2^ values and horizontal axis indicates the minor allele frequencies of the SNPs. Sample regions analyzed are indicated at the top of each panel

To assess the improvement in imputation accuracy gained by adding haplotypes from a different population, genotype imputation accuracies were compared among different reference panels as follows: 1KJPN consisting of 1070 Miyagi samples; 1KJPN+ consisting of 1KJPN and the three other Japanese populations analyzed in this study; and 1KGPp3, the phase 3 panel of the 1000 Genome Project (Fig. 4). Imputation accuracy with the 1KJPN+ panel was better than that with the 1KJPN panel and the improvement was prominent for rare variants. The r^2^ of rare variants with 1KJPN+ improved to 0.7269, 0.7485, 0.6639, and 0.7037 for Miyagi, Iwate, Nagahama, and Aki, respectively, compared with the r^2^ obtained with 1KJPN. For the three reference panels, the imputation coverage (proportion of variants with confidence: imputation r^2^ ≥ 0.8) across different non-reference allele frequency bins as well as the number of variants that were efficiently imputed are shown in Additional file 3: Table S2. Our results suggest that the addition of samples from other parts of Japan is necessary to further improve 1KJPN as a reference panel for the entire Japanese population. On careful examination, we found several flip-flops occurred between 1KJPN and 1KJPN+ for alleles with high frequencies; for example, Nagahama samples with MAFs around 0.050 (Fig. 4). This may be caused by the design of 1KJPN, which targets the imputation of SNPs with relatively low frequencies (MAF ≥0.5%). [11].

### SNPs that differentiate between Miyagi and Nagahama or Aki

The efficiency of 1KJPN as a reference panel for genotype imputation for most Japanese has been shown, [9] and is considered to sufficiently cover SNPs with about 1% MAFs in the Japanese population. However, two medically relevant questions remain unanswered, namely, how many biologically relevant SNPs exist among populations in the various regions of Japan, and how different are the MAFs of critical SNPs among Japanese from different regions where the populations might have different genetic disease susceptibilities? To answer these questions, we listed the SNPs that segregate in 1KJPN and exhibit statistically significant differences (*p* < 10^− 7^) in allele frequencies between Miyagi and Nagahama or Aki (Table 2). We detected fewer than 10 SNPs that were present in the Nagahama or Aki population but absent in the Miyagi population (1KJPN), and these SNPs had no exonic variants (Table 2).

**Table 2:** SNP_S_ that differentiate between Miyagi and Nagahama of Aki based on MAF differences

test	rsid (dbSNP 138)	Chr	Pos (hg19)	Miyagi	Nagahama	Aki	p-value	Annotation
Nagahama-Miyagi	rs1899621	3	157280172	0.03178	0.1795	0.0625	4.44.E-07	PQLC2L: Intronic
Nagahama-Miyagi	rs9501875	6	2685970	0.09331	0.2949	0.04688	8.41.E-07	MYLK4: Intronic
Nagahama-Miyagi	rs148081741	6	135758259	0.02944	0.2051	0.01562	4.32.E-09	AHI1: Intronic
Nagahama-Miyagi	rs141380643	6	135812869	0.02897	0.2051	0.01562	3.54.E-09	AHI1: Intronic
Nagahama-Miyagi	chr7:144127237:C:T	7	144127237	0.0323	0.1795	0.1406	5.31.E-07	intergenic
Nagahama-Miyagi	rs55897843	10	28739479	0.01731	0.1538	0	4.98.E-08	LOC105376468: Intronic
Nagahama-Miyagi	chr11:32314153:G:A	11	32314153	0.03087	0.1795	0.09375	3.26.E-07	intergenic
Nagahama-Miyagi	chr16:47678044:A:T	16	47678044	0.01457	0.1282	0.0625	7.88.E-07	PHKB: Intronic
Nagahama-Miyagi	rs8094961	18	14267309	0.009615	0.1154	0	3.69.E-07	intergenic
Nagahama-Miyagi	rs57064200	21	46286788	0.3396	0.6282	0.371	3.86.E-07	PTTG1IP: Intronic
Aki-Miyagi	rs117933761	1	100267335	0.01402	0.03846	0.1562	8.90.E-08	intergenic
Aki-Miyagi	rs4922078	8	19512537	0.3312	0.359	0.6406	7.15.E-07	CSGALNACT1: Intronic
Aki-Miyagi	rs9423657	10	5607370	0.02682	0.03846	0.1875	3.33.E-07	LOC105376381: Intronic
Aki-Miyagi	rs10899501	11	78131408	0.4565	0.4359	0.1452	3.96.E-07	intergenic
Aki-Miyagi	chr13:103019948:C:T	13	103019948	0.0565	0.1923	0.25	7.78.E-07	FGF14: Intronic
Aki-Miyagi	rs118020607	14	99987537	0.06232	0.1026	0.2656	5.24.E-07	CCDC85C: Intronic
Aki-Miyagi	rs59993898	15	80826474	0.009813	0.01282	0.125	8.60.E-07	ARNT2: Intronic
Aki-Miyagi	rs150711498	18	21327345	0.008879	0.01282	0.125	4.66.E-07	LAMA3: Intronic
Aki-Miyagi	rs11669387	19	33999497	0.1076	0.141	0.3438	7.66.E-07	PEPD: Intronic

Deep-coverage WGS (approximately 30×) of an individual genome can detect very rare variants that were not identified previously. We collected the SNPs not found in 1KJPN (Miyagi population) but found multiple times among the Iwate, Nagahama, and Aki populations (Table 3). The average numbers of such non-1KJPN SNPs were higher in the Aki and Nagahama populations than in the Iwate population, which corresponds to the F_ST_ data (Table 1, Fig. 1c). This indicates that the addition of samples from distant areas was more effective for collecting rare variants than the addition of samples from neighboring areas. Among the rare variants, functionally important SNPs that caused amino acid changes or premature stop codons were very rare (0.05 to 0.26 SNPs per genome). The data indicate that there are few deleterious mutations in Japanese from other parts of the country that are not found in 1KJPN. In other words, 1KJPN may be sufficiently comprehensive for genotype imputation for most genome-wide association studies on the population of mainland Japan.

**Table 3:** Numbers of SNPs found in three populations but not found in 1KJPN (Miyagi population)

Type	Iwate (per person)	Nagahama (per person)	Aki (per person)
Total SNP_S_ (AC > =2)*	113,541 (834.86)	33,067 (847.87)	44,634 (1275.26)
Exonic	1596 (11.74)	411 (10.54)	593 (16.94)
Mis sense	1032 (7.59)	282 (7.23)	366 (10.46)
Stop gain	14 (0.10)	2 (0.05)	9 (0.26)

*AC* allele counts in the three regions

## Discussion

We evaluated the influence of genetic diversity on the accuracy of genotype imputation among populations from different parts of Japan. Previous studies reported clear genetic differentiation between individuals from Okinawa (Ryukyu area) and individuals from the rest of Japan (Hondo), but genetic differentiation among local regions in the Hondo area has been reported to be very low and not to show distinct clusters. [15, 16] In this study, however, we found genetic clusters separated in accordance with geographic location (Miyagi, Iwate, Nagahama, and Aki) using a haplotype-based statistical method [14] (Fig. 1b). Among these areas, genetic diversity was shown to be correlated with geographic distance; for example, the Miyagi and Iwate populations were genetically closer than any other pair of areas. The differences in imputation accuracy with the 1KJPN panel among samples from these regions (Fig. 3) were also consistent with this diversity. Because the 1KJPN panel contains samples only from the Miyagi area, more haplotype segments were shared with this area than with other regions. Notably, the imputation accuracy of the Iwate samples was very close to that of the Miyagi samples, even though the Iwate samples are not included in the 1KJPN panel. This is consistent with another report showing that genetic similarities among subpopulations were correlated with geography on the Japanese archipelago. [13] These observations support the idea that ancestry matching between the subjects of genotype imputation and the donors of genomic data for a whole-genome reference panel is effective for improving imputation accuracy, especially for low-frequency and rare variants.

We demonstrated that haplotypes specific to samples in a local area had substantial impact on imputation performance, especially for low-frequency and rare variants. As mentioned above, genetic differentiation within the Hondo population was small in terms of SNP frequency, but apparent when haplotype sharing between samples was considered. Because imputation algorithms essentially rely on haplotype sharing between the reference panel and the study samples, the genetic differentiation between 1KJPN and other Japanese regions might have been substantial. Our results show that the imputation accuracy for common variants was only marginally affected by the combination of the panel and the area (Fig. 3), and by the addition of region-specific haplotypes to the panel (Fig. 4), suggesting that the common haplotypes contained in the 1KJPN panel cover the haplotype diversity of the Hondo area. However, the imputation accuracy of low frequency and rare variants improved when area-specific haplotypes were added to the 1KJPN panel. This means that long-persisting yet rare haplotypes may exist in each area, and that the imputation accuracy can be improved when matching the haplotype panel and samples in that area. These results provide important information for future extension of haplotype reference panels in population cohorts.

## Conclusions

Our data suggest that 1KJPN can cover most of the variants found in the samples from other areas in the Japanese mainland outside of Miyagi and that 1KJPN can be used as a representative of the Japanese population’s genomes, making it is useful genome reference panel for other parts of the Japanese mainland. We also showed that the addition of samples not included in 1KJPN improved imputation efficiencies for SNPs with MAFs under 1% from most of the Japanese archipelago.

## Methods

### Sample preparation

The haplotype reference panel (1KJPN panel) was constructed from the whole-genome sequences of 1070 participants from the prospective cohort study of the TMM project. [9] All samples in this panel were obtained from individuals recruited in the Miyagi Prefecture. In the present study, we added samples from individuals recruited in the Iwate Prefecture to our cohort, as well as samples from age- and sex-stratified random samples from external cohorts for comparison, namely, the Nagahama study [17, 18] and the Aki area from the JPHC-NEXT study [19]. WGS and SNP array genotyping were conducted for 136, 39, and 36 samples from the Iwate, Nagahama, and Aki cohorts, respectively. Participants from these cohorts provided written informed consent to undergo WGS in the collaboration studies. For the WGS, 2.5 μg of DNA was dissolved in TE buffer (Tris pH 8.0 10 mM, EDTA 1 mM) or distilled water (100 ng/μL). Aliquots of 500 ng were prepared for the SNP array analyses. Detailed WGS methods followed previous studies. [9, 20] SNP array genotyping was performed using Japonica arrays (Toshiba Corporation, Tokyo, Japan).

### Construction of haplotype reference panel

The haplotype reference panel was constructed from WGS data. [9] We constructed an extended haplotype reference panel (1KJPN+ panel) consisting of newly sequenced samples from Iwate, Nagahama, and Aki cohorts using the method described previously, [9] in addition to the 1070 samples originally included in the 1KJPN panel. Variant calling with filtering and haplotype phasing were according to the methods used to construct the 1KJPN panel. [9] Briefly, read mapping and genotype calling were performed using Bowtie2 (version 2.1.0) [21] and Bcftools (version 0.1.17-dev) [22], respectively. Sequence depth criteria for filtering unreliable genotypes were determined on an individual bases to realize genotype concordance between next-generation sequencing variant call data and SNP array call data as 99.8%. We then phased the genotypes obtained from WGS using the SHAPEIT2 program (version 2.r644). [23] Cryptic relatives inferred through an identity by descent estimate (PI_HAT value > 0.125) were removed from the reference panel. PI_HAT values were calculated using the PLINK (version 1.9) program. [24]

### SNP array genotyping

Genotype calling was conducted using the apt-probeset-genotype program in the Affymetrix Power Tools suite (version 1.18.2; Thermo Fisher Scientific Inc., Waltham, MA). Quality control (QC) criteria were set in accordance with the manufacturer’s recommendations (dish QC ≥0.82; sample call rate ≥ 97%) and were met by all the samples. SNP-based QC was conducted using the Ps classification function in the SNPolisher package (version 1.5.2; Thermo Fisher Scientific Inc.). SNPs that were categorized as “recommended” by the Ps classification were retained. SNPs with call rate < 97.0%, Hardy–Weinberg equilibrium of *p* < 10^− 6^, or MAF < 0.5% were excluded from the downstream analysis.

### Population structure analysis

The SNP genotype data of the TMM samples were obtained by whole-genome sequencing or using the Japonica array. We obtained the corresponding SNP genotype data from next-generation sequencing analysis for the cases that were not analyzed with the Japonica array. To analyze the population genetics structure compared with that of East Asian populations, we downloaded the SNP data of chromosome 1 of unrelated East Asian populations (Dai Chinese, CDX; Han Chinese in Beijing, CHB; Han Chinese South, CHS; Tokyo Japanese, JPT; and Kinh Vietnamese, KVH) from the 1000 Genomes Project [25]. We selected the SNPs for which probes were included on the Japonica array [11] and used the VCFtools package to further filter the variants and individuals and the PLINK software package to calculate r^2^ scores. Indels and SNPs with maximum detection fraction > 0.1, smallest MAF 0.05, and maximum r^2^ 0.8 were filtered out. The calculation of principal components for the SNP genotype was performed using the PLINK package.

Weir and Cockerham’s F_ST_ value estimators [26] were calculated between all pairs of populations using PLINK. Based on the resultant F_ST_ matrix, a network was inferred among populations using the neighbor-net method [27] in the SplitTree program [28]. Sample clustering by haplotype sharing was performed with the fineSTRUCTURE program [14]. Haplotype phasing for this analysis was carried out using the SHAPEIT2 program (version 2.r644) with the default settings [23].

### Evaluation of imputation performance

We performed genotype imputation using the IMPUTE2 program. [29] Variants in the reference panel that had the same position in the Japonica array [11] (32,913 SNPs) were extracted for use in genotype imputation. The remaining variants in the panel (1,012,074 SNPs) were used to evaluate the accuracy of imputation of the true genotypes. Because the Miyagi samples used to test the reference panels are included in the reference panel (i.e., 1KJPN), we conducted a leave-one-out cross-validation experiment. Namely, each sample in the panel was extracted from the panel one after the other, and then genotype imputation of that sample was conducted against the entire panel without that sample. Because this procedure was repeated for all samples in the panel it required intensive computational resources, so the evaluation of imputation performance was conducted for SNPs only on chromosome 10 (1,044,987 SNPs). Through this process, we obtained imputed genotypes for every sample in the panel. Genotype imputation for the samples that were not included in the reference panel (i.e., Iwate, Nagahama, and Aki samples) was done with the the IMPUTE2 program. Imputation accuracy was measured using Pearson’s correlation coefficient (r^2^) between true genotypes, taking a value of 0, 1, or 2, and imputed genotype dosages with values between 0 and 2. The r^2^ values were estimated upon aggregating the variants in the reference panel that were stratified by non-reference allele frequency to visualize the imputation accuracies for rare SNPs. These evaluations were conducted on the SNPs that were identified in all the examined reference panels.

## Additional files

Additional file 1: **Table S1.** The imputation coverage (proportion of variants with an imputation r2 ≥ 0.8), with each reference panel across different MAF bins. (XLSX 8 kb)

Additional file 2: **Figure S1.** Pairwise coincidence matrix of individuals from the four Japanese populations created by fineSTRUCTURE. Samples from Miyagi residents whose maternal grandmother was also born in Miyagi Prefecture (288 of the 1070 samples) were used to analyze haplotype sharing among the four Japanese populations. Color scale (left panel) represents the posterior confidence probability. Areas of each sample are shown by a colored box at the top of the figure. (PDF 312 kb)

Additional file 3: **Table S2.**The imputation coverage (proportion of variants with confidence: imputation r2 ≥0.8) across different non-reference allele frequency bins and the number of variants that were efficiently imputed. (CSV 20 kb)
