# Supplementary figures and images for: Regional genetic differences among Japanese populations and performance of genotype imputation using whole-genome reference panel of the Tohoku Medical Megabank Project
Source: BMC Genomics. 2018 Jul 24;19:551. doi: 10.1186/s12864-018-4942-0 (PMC6057088; doi:10.1186/s12864-018-4942-0)

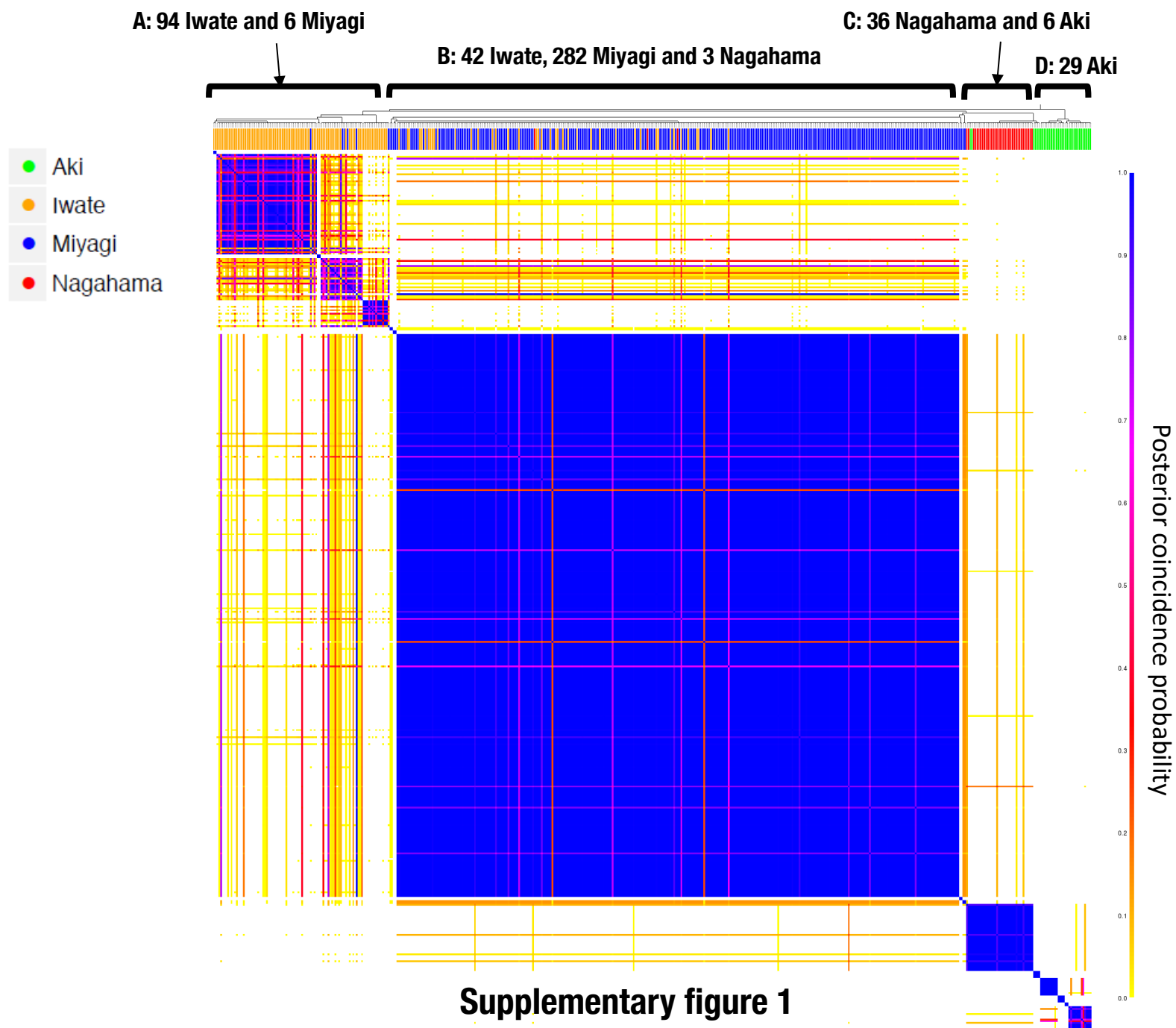

**Supplementary figure 1**

Supplement: Supplementary file 2 — Figure S1. Pairwise coincidence matrix of individuals from the four Japanese populations created by fineSTRUCTURE. Samples from Miyagi residents whose maternal grandmother was also born in Miyagi Prefecture (288 of the 1070 samples) were used to analyze haplotype sharing among the four Japanese populations. Color scale (left panel) represents the posterior confidence probability. Areas of each sample are shown by a colored box at the top of the figure. (PDF 312 kb) [file 12864_2018_4942_MOESM2_ESM.pdf]
